# Induction and Stabilization of Peptide Alpha‐Helices on Silica Nanoparticles

**DOI:** 10.1002/anie.8942173

**Published:** 2026-07-23

**Authors:** Fidel Lozano‐Elena, Seyed Amirabbas Nazemi, Patrick Shahgaldian, Sebastian Wendeborn

**Affiliations:** ^1^ Institute for Chemistry and Bioanalytics – School of Life Sciences University of Applied Sciences and Arts Northwestern Switzerland (FHNW) Muttenz Switzerland; ^2^ Swiss Nanoscience Institute Basel Switzerland

**Keywords:** ⍺‐helix stabilization, conformation selection, nanoparticles, peptides, reductive amination

## Abstract

Stabilizing secondary structure elements is critical to controlling peptide and protein bioactivity, stability, and function. The α‐helix, central to molecular recognition and structural integrity, is a particularly valuable target for stabilization in biotechnological and nanomaterial applications. However, non‐covalent peptide–nanomaterial interactions often lack robustness and reproducibility. We report a covalent immobilization strategy to induce and stabilize α‐helical conformations on amino‐functionalized silica nanoparticles (SNPs). This approach leverages dynamic covalent peptide–surface coupling to enable thermodynamic selection of α‐helical conformations. Alanine‐rich peptides incorporating periodic lysine residues were designed to enable covalent anchoring while promoting helix formation. Immobilization markedly enhances thermal stability compared to free peptides. Systematic variation of lysine positioning shows that terminal attachment induces helicity, whereas incorporation every second helical turn provides optimal conformational and thermal stability. Crucially, the helical face oriented away from the nanoparticle surface remains accessible for molecular recognition. Streptavidin‐binding peptides immobilized via this method retain their specific binding activity while exhibiting superior thermal robustness. This sequence‐guided approach establishes a robust framework for covalent peptide–nanoparticle conjugation, enabling precise secondary structure stabilization and functional biointerfaces for thermally stable protein–protein interaction platforms.

## Graphical abstract

Covalent docking turns silica nanoparticles (SNPs) into α‐helix‐stabilizing surfaces. Dynamic imine formation allows peptides with lysine residues aligned on one helical face to adapt to the surface before reduction locks the structure in place, while preserving the opposite face for functional recognition.

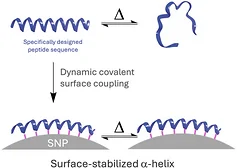

## 1 Introduction

The biological function of proteins is determined by their three‐dimensional architecture, with secondary structures of the amino acid chains constituting fundamental structural elements. These structures consist of regular folding patterns, such as α‐helices and β‐sheets, that are primarily stabilized by hydrogen bonds and dipole–dipole interactions between backbone atoms [1, 2]. In proteins, secondary structures mainly contribute to stability, while catalytic activity and molecular recognition elements are more often mediated by flexible, unstructured loops that can adapt their conformation in response to local stimuli [3]. In contrast, for shorter peptides, the formation of stable secondary structures is generally disfavored, as secondary structure stabilization typically requires longer sequences [4, 5, 6, 7]. At the same time, secondary structure is often more critical for peptide function (e.g., cell penetration and biological activity of peptides) [8, 9]. Since most of the residues in peptides are exposed to the solvent, their secondary structures are particularly sensitive to physical and chemical changes in the media, including temperature, pH, presence of cations, and hydrophobicity. This sensitivity has been leveraged for the development of *switchable* nanomaterials with diverse applications such as the self‐assembly of supramolecular structures [10, 11, 12], nanoparticle stabilization and aggregation [13, 14], drug delivery systems [15], therapeutics [16, 17], and advanced imaging tools [18].

Interactions between peptides and nanomaterial surfaces can induce the formation of peptides secondary structures, particularly α‐helices. For example, adsorption of specific peptides onto the surface of gold, silica or hydroxyapatite nanoparticle surfaces [19, 20, 21, 22, 23], and absorption into porous silica nanoparticles has been shown to stabilize peptides secondary structure [24]. As the induction and stabilization of these secondary structures are mainly mediated by ionic interactions, we postulate that a peptide's secondary structure stabilized by covalent bonds to a surface would lead to more robust secondary structures, with higher thermodynamic stabilities compared to secondary structures stabilized by non‐covalent interactions.

In this work, we demonstrate that rational design of alanine‐rich peptide sequences containing strategically positioned lysine residues enables covalent immobilization of peptides on the surface of silica nanoparticles (SNPs) while inducing and stabilizing an α‐helical conformation. This strategy exploits reversible imine bond (Schiff base) formation between lysine side chains and amine‐functionalized SNPs, allowing dynamic peptide–surface covalent reactions enabling thermodynamic conformation selection. Reductive amination can irreversibly “lock in” the resulting α‐helical structure. The underlying chemistry, widely applied for protein immobilization on SNPs, is based on glutaraldehyde‐mediated imine formation followed by reductive amination [25, 26, 27]. Here, we deliberately exploit its dynamic, reversible character to promote multivalent attachment (multiple lysine residues) of individual peptide molecules to the APTES lattice on the SNP surface, resulting in the formation of the α‐helix. Furthermore, we show that the solvent‐exposed face of these SNP‐bound peptide secondary structures can be engineered to enable protein binding with increased thermostability.

## 2 Results and Discussion

### 2.1 Alanine‐Rich Peptides Design and Synthesis

**FIGURE 1:**
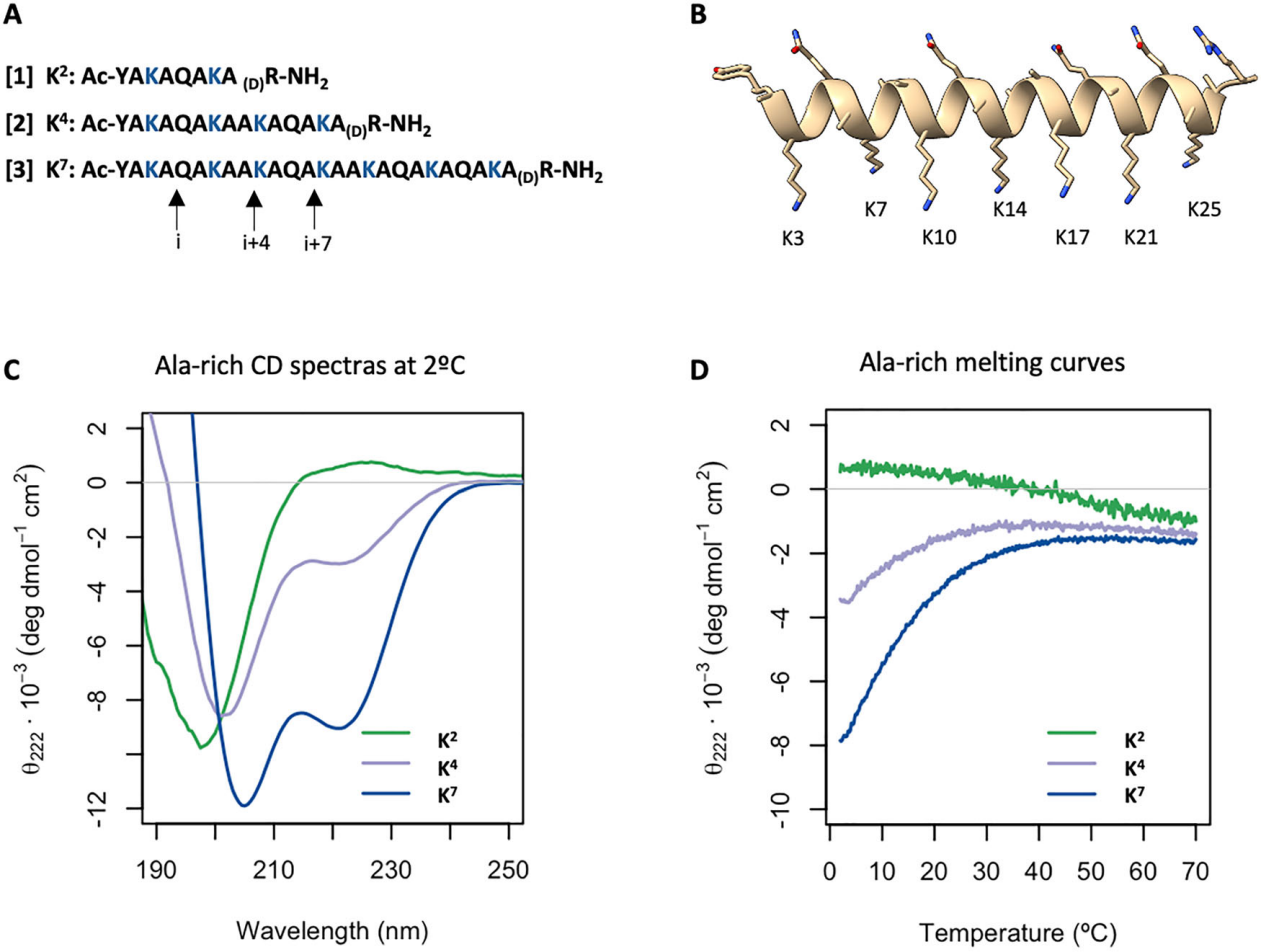
Alanine‐rich peptides form α‐helices in solution at low temperature. (A) Sequence of the Ala‐rich peptides. One lysine is introduced in every turn of the α‐helix, at the position i, i+4, i+7, i+11, i+14, etc. (B) Model of the longest Ala‐rich peptide, K^7^ [**3**], designed to expose all lysine side chains to the same side of an α‐helix. (C) CD spectrum of K^7^ peptide, [**3**], in solution. (D) Ellipticity at 222 nm of the three Ala‐rich peptides in function of increasing temperature.

To investigate the possibility of inducing and retaining an α‐helical structure by covalently immobilizing a peptide onto SNPs, we designed model peptides based on an alanine‐rich sequence (Ala‐rich), which are known to favor α‐helix formation [28, 29]. We designed the peptides such that Lys side chains (i.e., surface attachment points) are positioned on the same face of the α‐helix. This conformation is expected to be “locked‐in” once the lysine side chains are covalently and irreversibly bound to the surface of the nanoparticle. We designed three Ala‐rich model peptides of varying lengths: a 9‐mer [**1**] (K^2^), a 16‐mer [**2**] (K^4^) and a 27‐mer [**3**] (K^7^)—each containing 2, 4 and 7 Lys residues, respectively (Figure 1A), all expected to be positioned on the same face once the α‐helix is formed (Figure 1B). Peptide naming, e.g., K^7^, follows a face‐projection notation, in which superscripts denote the number of residues contributing to one α‐helical face. These peptides were acetylated at their terminal NH_2_ to avoid its inclusion as an additional attaching point. We also included design principles from other Ala‐rich peptides previously described: [22] (i) addition of a D‐Arginine residue at the C‐ terminus of the peptide to provide a capping motif that further stabilize the α‐helix [30]; (ii) including a tyrosine residue as a spectrophotometric probe [22]; and (iii) adding glutamine, (Gln) residues opposite the Lys‐rich face of the α‐helix to avoid the formation amphiphilic peptides and reduce aggregation [22, 31] (Figure 1B). The designed peptides were produced by solid‐phase peptide synthesis and characterized by circular dichroism (CD) spectroscopy. In solution, α‐helix formation in these Ala‐rich peptides is, as expected, favored at low temperature [6, 22]. Consistently, the circular dichroism (CD) spectrum of K^7^ peptide, [**3**] (27‐mer), at 2°C, exhibits the characteristic features of an α‐helical conformation, [32] including a minimum negative value of ellipticity (θ) at λ = 222 nm, and positive values at λ<193 nm (Figure 1C). The α‐helix content at 2°C was estimated to be 59.1% (47.9% regular + 11.2% distorted helix, as for the SELCON3 algorithm [33, 34], see methods, Table S1). This helical content decreased rapidly with increasing temperatures (only 13.0% α‐helix content at 50°C, Figure 1C). Regarding the shorter versions of the Ala‐rich peptides, only K^4^ [**2**] (16‐mer) showed α‐helix features at low temperature. However, the negative ellipticity at 222 nm, a hallmark of α‐helix structure, is lost faster than in the K^7^ peptide [**3**] (Figures 1D, S1). This behavior is consistent with the greater propensity of longer peptides to form secondary structures [6]. Consequently, we continued the study with the longest version of the Ala‐rich peptide, K^7^ [**3**]. Additionally, we found that alkaline pH (>10) dramatically induces the α‐helix structure in our model peptide (Figure S2). We attribute this effect to reduced electrostatic repulsion between neighboring Lys residues at higher pH, where Lys (*pK_a_* ≈ 10.5) is less protonated and therefore less positively charged. At physiological pH, this repulsion is likely unfavorable, particularly because lysine residues in an α‐helix are oriented toward the same face of the peptide, therefore destabilizing this alignment. We leveraged this pH‐dependent behavior to pre‐form the α‐helical structure prior to immobilization on the SNP surface at higher pH (10.8).

### 2.2 Peptide Immobilization Onto Silica Nanoparticles Induces and Stabilize α‐Helix Formation

**FIGURE 2:**
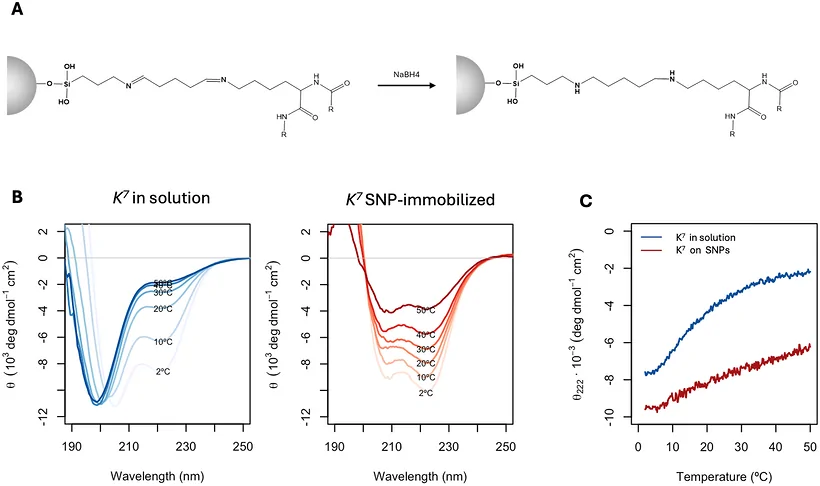
Immobilization of peptides onto SNPs surface induces and stabilizes the α‐helix secondary structure. (A) Chemical strategy used to immobilize peptides. Amino groups on the SNP surface react first with glutaraldehyde, then the peptide is added to react with the exposed aldehyde functions. At this stage, equilibration of imine bond formation is possible, and finally, the imine bonds are reduced using NaBH_4_. (B) Ellipticity of K^7^ peptide **[3]** in solution at different temperatures (left) compared with the immobilized counterpart (right). (C) Thermal stability of the K^7^ **[3]** α‐helix in solution (blue) and after immobilization (red), as measured by the ellipticity at 222 nm as a function of temperature.

Peptides were covalently immobilized onto 3‐aminopropyltriethoxysilane (APTES)‐functionalized SNPs (SNP‐NH_2_) with a diameter of 48 ± 5 nm (as expected, no significant change in particle size was observed after APTES functionalization, as confirmed by scanning electron microscopy; Figure S3) using glutaraldehyde, a dialdehyde that links surface amines of the SNPs to Lys side‐chains amines through reversible imine bond formation. This coupling chemistry is selective for primary amines, and because the peptide *N*‐terminus was capped as an acetamide, immobilization is expected to occur through the Lys side‐chain amines rather than through other amino acid residues. This initial step is dynamic in nature, allowing continuous bond formation and exchange and enabling equilibration of peptide–surface interactions. After this equilibration period (30 min), the formed imines were converted to stable secondary amines by reductive amination with NaBH_4_ (Figure 2A), thereby irreversibly fixing the peptide in its surface‐bound conformation. Peptide immobilization efficiencies ranged between 70% and 80%, as quantified by tyrosine absorbance (λ_280_) of unreacted peptide in the reaction mixture liquid phase. Importantly, we found that elevated pH not only maximized the α‐helical content of the model peptides at low temperature prior to immobilization but also markedly increased the immobilization efficiency (Figure S4). The best results were obtained when peptides were reacted under alkaline conditions (pH 10.8, 10 mM carbonate buffer) and at low temperature (4°C), to pre‐form the α‐helix in solution. Following immobilization, the peptide‐derivatized SNPs were resuspended in buffer at pH 7 prior to CD analysis (see methods). The CD spectra of immobilized K^7^ peptides [**3**] show an increased α‐helical content at 2°C (75.5%, Table S1) and, importantly, showed markedly enhanced thermal stability of the α‐helix compared to the peptide in solution (Figure 2B,C). At 50°C, the immobilized peptide showed a total of 56.9% of α‐helical content vs. 13% of the same peptide in solution, Table S1). Mixing peptides with the same concentration of APTES‐functionalized SNPs, in the absence of covalent immobilization, neither enhanced α‐helix formation and stability nor interfered with CD spectra measurement, confirming that the SNP surface alone does not induce α‐helix formation (Figure S5). In addition, peptides immobilized at lower pH values (4.0, 7.0 and 9.2), showed lower content of α‐helix structure after immobilization on SNPs and resuspension at neutral pH (Figure S6), confirming the beneficial effect of α‐helix pre‐formation on later α‐helix stability. To rule out that the observed stabilization arises from glutaraldehyde‐stappled peptides, we analyzed the liquid fraction of the reaction mixture by mass spectrometry. The results show no mass shift on the non‐immobilized peptide, representing ∼25% of the initial peptide concentration used in the reaction, and confirming the absence of stapled peptides (Figure S7). Together, these results support a mechanism in which dynamic covalent peptide–surface interactions promote α‐helix formation, which is subsequently stabilized upon irreversible immobilization. Of note, SNP‐only controls gave flat CD spectra centered around 0 mdeg after baseline correction, confirming that the nanoparticles did not introduce an apparent CD signal. For the ∼50 nm SNPs used here, scattering effects were limited, and all suspensions remained stable over the CD acquisition time, with no visible precipitation or sedimentation.

Finally, to evaluate the versatility of our strategy, we explored the use of other dialdehydes for peptide immobilization, aiming to reduce the flexibility provided by the glutaraldehyde linkers. Oxaldehyde (*glyoxa*l) and 1,4‐benzenedicarboxaldehyde (*terephtalaldehyde*, TPA) were tested, both resulting, with marginal differences, in a clear induction and stabilization of α‐helical structure (Figure S8). However, the extent of stabilization was not markedly different from that achieved using glutaraldehyde (Figure S8), which may be explained by the intrinsic flexibility of the aminopropyl chains of APTES.

**FIGURE 3:**
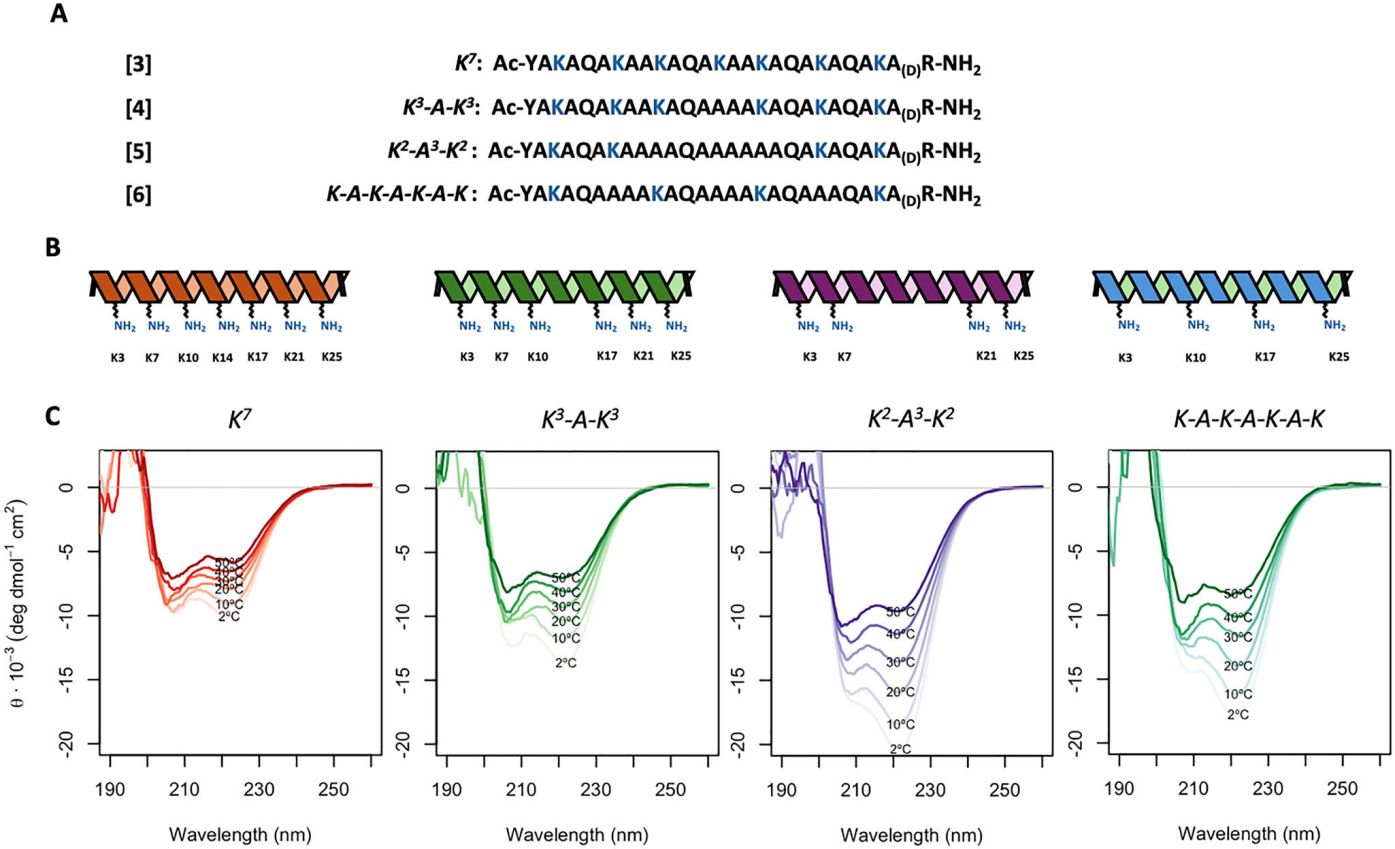
Peptide variants reveal the sequence requirements for optimized α‐helix formation and stabilization. (A) Sequences of the variants for the K^7^ peptide synthetized and immobilized, (B) Representation of the putative α‐helixes formed by the Ala‐rich peptides showing the orientation of Lys side chains, serving as attaching points: K^7^ **[3]**, seven attaching points, one every turn (red); *K^3^‐A‐K^3^* **[4]**, missing one attaching point in the middle (green); *K^2^‐A^3^‐K^2^* **[5]**, missing three attaching point in the middle (purple); and *K‐A‐K‐A‐K‐A‐K* **[6]**, with only one attaching point every two helix turns (blue). (C) CD spectra at different temperatures of the peptides variants immobilized on SNPs.

Next, we investigated the minimal attachment‐point requirements required to stabilize the α‐helical conformation. To this end, we designed and synthesized a series of variants for the *K^7^* [**3**] model peptide in which one or more of the seven Lys residues were removed and substituted with Ala: *K^3^‐A‐K^3^* [**4**], missing the central Lys; *K^2^‐A^3^‐K^2^* [**5**], missing the three central Lys, and *K‐A‐K‐A‐K‐A‐K* [**6**], containing only one lysine every two helical turns (Figure 3A,B). In this latter design, the peptide is expected to adopt an α‐helical conformation in which the Lys side chains are nearly aligned, occupying the same angular position in the helical wheel. Immobilization onto SNPs of all peptide variants resulted in an induction of the ⍺‐helix, as indicated by their CD spectra (Figure 3C) compared to ⍺‐helix formation of these variants in solution (Figure S9A). In contrast to the original design featuring one Lys per helical turn, *K^7^* peptide [**3**], the CD spectra of these variants indicate that removal of a single attachment point in the central region of the helix has minimal impact on the overall secondary structure, while slightly increasing the helical content. (Figure 3C, *K^3^‐A‐K^3^* [**4**] design). Removing the three central Lys, thereby restricting immobilization to the peptide termini, resulted in substantially higher ⍺‐helical content at low temperature, but this comes at the cost of reduced thermal stability (Figure 3C, *K^2^‐A^3^‐K^2^* **[5]** design). Consistent with this observation, incorporating Lys only every two turns also increases the helical content at lower temperatures, but, again, at the expense of thermal stability (Figure 3C, *K‐A‐K‐A‐K‐A‐K* [**6**] design). Taken together, these results reveal that the number and position of attaching points, that is, Lys, both play a key role in balancing maximum helical content with thermal stability (Figure S10). We speculate that this behavior arises from reduced electrostatic repulsion between charged Lys residues, as they are further apart than in the *K^7^* **[3]** peptide. This may allow more efficient folding of the peptide prior to immobilizing on the SNPs surface. Consistent with this interpretation, the secondary structure of these peptides exhibits a reduced dependence on pH (Figure S9B). The higher flexibility of these variants likely accounts for their better folding at low temperatures, but also for their decreased thermal stability. Importantly, the immobilization at the extremities of the sequences, in combination with a flexible, non‐immobilized peptide stretch in the middle of the sequence (*K^2^‐A^3^‐K^2^* **[5]** design), seems to favor the ⍺‐helix formation further, although it progressively unfolds with temperature, likely because the unrestricted stretch loses the secondary structure. In addition, in the case of the *K‐A‐K‐A‐K‐A‐K* **[6]** variant, Lys residues are perfectly oriented toward one side of the ⍺‐helix, likely also contributing to the high helicity observed at low temperatures. Notably, these designs, [**5**] & [**6**], appear to provide a more favorable balance between helix formation and surface attachment than the original *K^7^* peptide [**3**]. The original design forms a more stable structure, but the nature of the seven attachment points may compromise an ideal α‐helix formation. As a result, a slightly distorted, yet thermally more stable, α‐helical structure is observed. The *K^2^‐A^3^‐K^2^* [**5**] & *K‐A‐K‐A‐K‐A‐K* [**6**] designs, on the other hand, will form a less thermally stable ⍺‐helix, but the inherent flexibility resulting from only four attachment points allows a more ideal α‐helical conformation.

### 2.3 Stabilized ⍺‐Helices Enable the Design of Functional Interfaces

**FIGURE 4:**
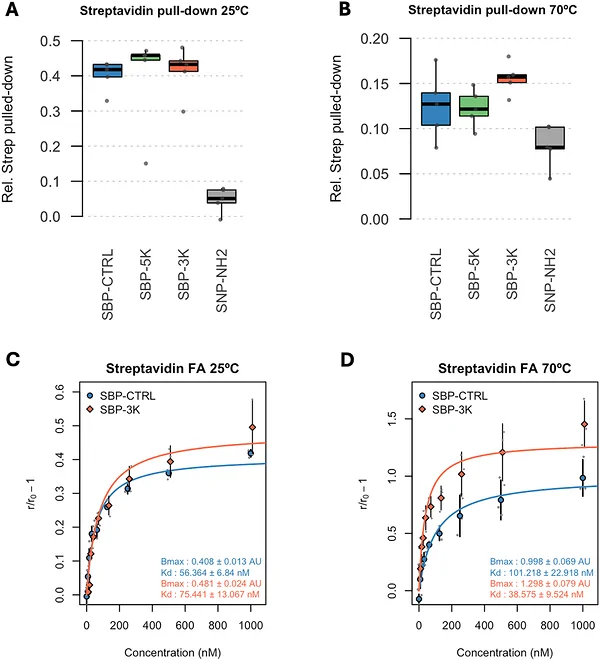
SNPs‐immobilized peptides retain function. Str‐binding peptides engineered to be immobilized and stabilized in an α‐helix conformation retain their binding capacity, as revealed by pull–down experiments and fluorescence anisotropy (FA) measurements. (A) Binding of Str to SNP‐immobilized peptides, as measured by the fraction of Str‐FITC remaining in the supernatant once incubated with the peptide‐SNP and spun down. (B) Same as in A, but Str‐FITC and peptide‐SNP were incubated at 70°C prior to spinning down. (C) Binding of Str to SNP‐immobilized peptide as measured by FA. (D) Same as in C but plates were incubated at 70°C before FA measurements. Note the increase in maximum FA signal and the better apparent affinity of the engineered SBP variant.

Next, we assessed whether immobilized peptides could still maintain a functional surface—for example a protein binding motif—on their solvent exposed side, opposite to the SNP–peptide interface (i.e., the side of the ⍺‐helix opposite the poly‐Lys region). To test this, we employed a Streptavidin‐Binding Peptide (SBP) immobilized on SNPs as a model system [35, 36, 37]. The SBP‐Tag binds streptavidin with low nanomolar affinity and, importantly, adopts an almost ideal α‐helical conformation upon binding, as revealed by crystallographic studies (Figure S11) [37]. We engineered a shorter, 25‐mer variant of the SBP‐Tag (NH_2_‐GHVVE **G** LA **G** EL **E** QLR **A** RL **E** HHPQFQ‐COOH), named SBP‐Tag2, that keeps the nanomolar binding affinity [37], and strategically introduced Lys residues for immobilization and helical stabilization (Figure S11). Two designs were studied: (i) a five‐Lys variant with one Lys per helical turn (G6K, G9K, E12K, A16K, E19K), named SBP‐5K [**7**], and (ii) a three‐Lys variant with one Lys every two helical turns (G6K, E12K, E19K), named SBP‐3K [**8**], which would be analogous to the *K‐A‐K‐A‐K‐A‐K* design [**6**]. As a control, we generated a variant with a single lysine substitution in the middle of the helix (E12K), SBP‐CTRL [**9**], allowing SNP attachment but without significant helical stabilization. Although all peptides showed marginal α‐helix features in their CD profiles at 2°C and 40°C, an indication of suboptimal helix formation in solution, immobilization on SNPs induced formation and stabilization of an ⍺‐helix in the engineered variants, as expected (Figure S12). We examined whether immobilized peptides retained their ability to bind streptavidin (Str). To this end, we incubated the peptide‐SNPs with fluorescein isothiocyanate (FITC)‐labeled‐Str and quantified the fluorescence remaining in the supernatant after centrifugation. Immobilized SBP peptides bound approximately 40%‐45% of Str (Figure 4A, 200 nM Str‐FITC and 200 nM immobilized peptides), demonstrating that peptide functionality, that is, protein binding, was retained after immobilization. This indicates that one interface of the immobilized helix remains accessible for binding. No major differences were observed between the control SBP (with only one Lys) and the engineered SBP variants. Unspecific binding to SNP accounted for less than 10% of the Str captured by the peptide‐modified SNPs (Figure 4A, “*SNP‐NH_2_”*). We next challenged peptide–streptavidin binding by thermally destabilizing the peptide secondary structure. After incubating the samples at 70°C prior to centrifugation, Str binding dropped to ∼12%. Notably, a modest increase in binding of the “three‐lysine” engineered variant, SBP‐3K **[8]**, compared to the control was observed (Figure 4B), suggesting that helix stabilization may confer an advantage under suboptimal binding conditions.

We further reasoned that the binding of Str to SNPs would drastically increase its hydrodynamic radius, thereby increasing its rotational correlation time and, consequently, its fluorescence anisotropy (FA). To probe this effect, FA of FITC‐labeled Str was measured as a function of peptide–SNP concentration. As expected, FA increased with increasing peptide‐SNP concentrations, confirming binding. Control and engineered peptides showed very similar apparent binding affinities, with equilibrium dissociation constants (*K_D_*) of 56.4 ± 6.8 nM and 75.4 ± 13.1 nM for control **[9]** and three lysine engineered variant [**8**], respectively (Figures 4C, S13). Although these affinities fall within the low nanomolar range, they are roughly an order of magnitude weaker than previously reported values [37]. This discrepancy is likely due to suboptimal peptide conformations upon SNP immobilization and/or steric hindrance between the SNP and Str as well as that caused by the FITC label on Str.

Consistent with the pull–down experiments, increasing the temperature revealed differences between the variants. The SBP‐3K [**8**] engineered peptide exhibited a 30% higher FA maximum signal and retained stronger affinity compared to the control peptide (Figures 4D, S12). These results suggest that a larger fraction of the control peptide becomes inactive at elevated temperatures, likely due to a higher TΔS contribution from loss of ⍺‐helix structure and increasing tumbling, rendering it unable to bind Str. In contrast, the SNPs‐immobilized SBP‐3K [**8**] engineered peptide retained a higher capacity to bind streptavidin. Together, these Str‐binding experiments demonstrate that immobilized peptides retain the functions associated with the residues placed on the face of the α‐helix that is not involved in the SNP binding. However, the engineered SBP peptides **[7]** & **[8]**, provided only modest improvements over immobilized control [**9**]. We attribute this to the imperfect α‐helix pose of the SBP‐Tag2 bound to streptavidin [37], as well as to the imperfect helices formed upon immobilization through several lysine residues, which likely do not align optimally with the required binding pose. In that sense, the control peptides [**9**] allow for a binding‐induced fit not possible in the engineered versions. Nonetheless, the engineered variants showed detectable functional advantages under challenging conditions. We therefore speculate that in a system requiring longer and/or more ideal α‐helices, the stabilizing effect of SNP immobilization should become substantially more pronounced.

## 3 Conclusion

The integration of peptides into nanomaterials is increasingly relevant in areas such as supramolecular assembly, imaging, and drug delivery systems. Peptide secondary structures, particularly ⍺‐helices, are key for these applications. Although the induction and stabilization of peptide structures by nanomaterials have been reported, it has so far been achieved mainly through ionic interactions. Here, for the first time, we demonstrate that ⍺‐helical conformations can be both induced and stably maintained, through covalent immobilization on nanoparticle surfaces. Our approach relies on peptides containing Lys residues positioned to face the same side of the prospective α‐helix. Basic pH and low temperature promote α‐helix formation, aligning the lysine side chains so they can react with aldehyde‐functionalized SNPs when the peptide's helical conformation is favored. We conclude that the optimal design for such a peptide does not place a lysine on every turn of the helix, instead placing one lysine every second turn results in a better aligned lysine pattern that also more closely matches with the aldehyde density SNP surface. The Lys pattern can nevertheless be tuned without losing the overall stabilizing effect, enabling control over a key trade‐off: fewer Lys yield more flexible peptides that form nearly perfect helices at low temperature but with less thermal stability, whereas more lysine residues produce more rigid, slightly distorted helices but are more stable at elevated temperatures. Finally, the solvent‐exposed side of the α‐helix remains available for incorporating functional sequences, as demonstrated with a functioning streptavidin binding motif on the surface. Extending this approach to more complex bioactive peptides will benefit from careful Lys placement, or from orthogonal protection/coupling strategies, to preserve both the designed SNP‐facing anchoring pattern and the solvent‐exposed functional interface. The immobilized α‐helices thus serve as a rigid scaffold onto which secondary‐structure‐dependent peptide functionalities can be engineered.

## Author Contributions

**Fidel Lozano‐Elena**: investigation, validation, data curation, visualization, writing – original draft, writing – review and editing, methodology, formal analysis, conceptualization, software. **Seyed Amirabbas Nazemi**: investigation, methodology, data curation, writing – review and editing, visualization, writing – original draft, validation, formal analysis, software, conceptualization. **Patrick Shahgaldian**: conceptualization, writing – review and editing, investigation, writing – original draft, methodology, visualization, validation, data curation, supervision, project administration, formal analysis, resources. **Sebastian Wendeborn**: conceptualization, investigation, writing – original draft, methodology, validation, visualization, writing – review and editing, data curation, supervision, formal analysis, project administration, resources.

## Conflicts of Interest

The authors declare no conflicts of interest.

## Supporting information



The authors have cited additional references within the Supporting Information [38, 39]. **Supporting File**: anie73752‐sup‐0001‐SuppMat.docx.

## Data Availability

The data that support the findings of this study are available from the corresponding author upon reasonable request.
