# Supplementary material for: Induction and Stabilization of Peptide Alpha‐Helices on Silica Nanoparticles
Source: Angew Chem Int Ed Engl. 2026 Jul 23;65(39):e8942173. doi: 10.1002/anie.8942173 (PMC13592319; doi:10.1002/anie.8942173)
Supplement: Supplementary file 1 [file ANIE-65-e8942173-s001.docx]

Supporting Information

**Induction and Stabilization of Peptide Alpha-helices on Silica Nanoparticles**

Fidel Lozano-Elena, Seyed Amirabbas Nazemi, Patrick Shahgaldian & Sebastian Wendeborn

**Table of content**

[Experimental procedures	2](#_Toc233623509)

[**Solid phase peptide synthesis (SPPS)**	2](#_Toc233623510)

[**Size exclusion chromatography (SEC)**	2](#_Toc233623511)

[**Silica nanoparticle preparation and functionalization**	2](#_Toc233623512)

[**Scanning electron microscopy (SEM)**	2](#_Toc233623513)

[**Peptide immobilization**	3](#_Toc233623514)

[**Circular dichroism (CD)**	3](#_Toc233623515)

[**Streptavidin binding and fluorescence anisotropy**	3](#_Toc233623516)

[Supplementary figures and table	4](#_Toc233623517)

# Experimental procedures

**Solid phase peptide synthesis (SPPS)**

Peptides were synthetized using Fmoc strategy in a Liberty Blue (CEM corporation) peptide synthesizer, using Rink Amide AM resin as solid support, 10% piperidine as deprotection solution and 0.5 M ethyl cyano(hydroxyimino)acetate (Oxyma) and 0.5 M N,N′-diisopropylcarbodiimide (DIC) as activators. The resin was thoroughly washed with dimethyl formamide after synthesis. Peptides were cleaved from resin and deprotected by incubating 3h with trifluoroacetic acid (TFA): triisopropylsilane (TIS) cocktail (TFA:TIS:H2O, 95:2.5:2.5) and subsequently precipitated in cold diethyl-ether. Peptide's identity and purity were confirmed by MALDI-TOF mass spectrometry, after which peptides were resuspended in 10% acetic acid in H_2_O and lyophilized.

**Size exclusion chromatography (SEC)**

SEC analysis were performed on Angilent 1260 Infinity Inert HPLC system equipped with UV detection using an AdvanceBio SEC column (1.9 µm, 200 Å, 4.6x300 mm, P.N. PL1580-5201; MW range 2-700 kDa). The column was operated under isocratic conditions with 150mM sodium phosphate buffer (pH 7.0) filtered (0.22 µm) at a flow rate of 0.2 mL min-1 for 35min. Samples were injected at 5 µL and monitored at 214, 258, 260 and 280 nm. The column backpressure was approximately 130 ± 10 bar. Calibration was performed using a gel filtration standard (Bio-Rad #1511901).

**Silica nanoparticle preparation and functionalization**

Silica nanoparticles (SNP) were synthesized based on a protocol inspired by the Stöber method.^[38]^ In brief, in a round-bottom flask, was added 330 mL ethanol and 43 mL nanopure water followed by the addition of 2.5 mL ammonium hydroxide and equilibrated at 20 °C for 1 hour. Subsequently 25 mL tetraethoxysilane (TEOS) was added to the mixture and incubated for 21 hours at 400 rpm stirring. The resulted milky suspension was centrifuged at 14,000 g for 20 min and washed two times with ethanol and two times with nanopure water under same centrifugation conditions. The produced SNPs were resuspended in 100 mL nanopure water and stores at 4 °C. The SNPs were diluted in 400 mL H_2_O to obtain a suspension of [SNPs] = 3.2 mg/mL. (3-aminopropyl)triethoxysilane (APTES; 0.2, 1 and 2 mM) was separately added to the previous suspension, and the reaction mixture was stirred at 20 °C, 400 rpm for 90 minutes. Subsequently, the suspension was centrifuged at 20000 g for 15 minutes, and the supernatant was removed. The particles were resuspended in nanopure water and submitted to ultrasonic treatment. This washing step was repeated twice. The particles produced, SNP-NH_2_ were then resuspended in 50 mL water and stored at 4 °C. Finally, to a suspension of SNP-NH_2_ (10 mL, 3.2 mg/mL) was added glutaraldehyde (25%, 40 μL). The reaction mixture was stirred at 20 °C, 400 rpm for 30 min. Then, the suspension was washed twice through centrifugation at 20000 g rpm and eventually resuspended in nanopure water to yield 40 mg/mL suspension.

**Scanning electron microscopy (SEM)**

Dispersions of silica nanoparticles (SNPs and SNP-NH_2_) in nanopure water were prepared and deposited onto silicon substrates. The samples were dried at room temperature under ambient conditions and subsequently sputter-coated with a Au/Pd for 15 s at 20 mA, resulting in a coating thickness of approximately 3 nm. Secondary electron micrographs were acquired using a field-emission scanning electron microscope (FE-SEM, JSM-IT800) operated in standard mode at an accelerating voltage of 10 kV and a magnification of 150,000 x. Particle size analysis was performed manually on the acquired micrographs using ImageJ (NIH), based on measurements of 250 particles, and the data were processed using OriginLab.

**Peptide immobilization**

Peptides were immobilized onto the glutaraldehyde-functionalized SNPs by mixing 10 mg/mL SNPs, 100 µM of peptide in 10 mM carbonate buffer pH 10.8 using a thermomixer at 4 °C. The mixture was incubated for, at least 30 min under shaking before adding 1.5 mM of NaBH4 to reduce imine bonds. Then, mixes were incubated overnight at 4 ºC under shaking. SNPs with immobilized peptides were centrifuged for 5 min at 3000 g. The supernatant was collected and the peptide concentration measured by spectrophotometry (λ280) to quantify the amount of peptide immobilized. SNPs were resuspended in an equal volume of 10 mM phosphate buffer pH 7 for CD measurements.

**Circular dichroism (CD)**

CD measurements were performed using a JASCO J-810 spectropolarimeter equipped with a Peltier module for temperature control and using a 1 mm path length quartz cuvette. Mean residue ellipticity (qRME) was obtained by first subtracting raw ellipticity of the blank (buffer) spectrum and then normalizing the final concentration of immobilized peptides, pathlength, and residue number[32]. For α-helix content estimation, the SELCON3 algorithm [33] was used (in its python version [34]), considering qRME between 195 and 240 nm and using SMP180 as reference set. [39]

**Streptavidin binding and fluorescence anisotropy**

To assess the binding of Str to the peptide-functionalized SNPs, a FITC-labeled Str (ACROBiosystems) was used. For pull-down experiments, 200 nM FITC-Str was mixed with 0.2 mg/mL peptides-SNPs (1µM immobilized peptide per mg/mL of 50 nm SNP) in PBS buffer pH 7.4 supplemented with 1% w/v BSA. Samples were incubated for 10 min shaking in a thermoblock at 25 ºC or 70 ºC, then centrifuged for 5 min at 20 000 g. A volume of 100µL supernatant was collected and transferred to black half-area 96-well plates (Greinier) for fluorescence measurements in plate reader (Biotek Synergy H1). For fluorescence anisotropy experiments, FITC-Str concentration was kept constant (Final concentration of 50 nM) and mixed with 2-fold dilution curve of SNPs-immobilized peptides (1 µM peptide per mg/mL of 50 nm SNPs). The assay was performed in PBS pH 7.4 with 1% w/v BSA, in black half-area 96-well plates (Greiner). Fluorescence Anisotropy was recorded with a plate reader equipped with polarizers (Biotek Synergy H1), using GFP filters (excitation 485 nm, emission 525 nm, slits 20 nm). Parallel and perpendicular fluorescence intensities were recorded and FA calculated as FA= (Iparallel - Iperpendicular) / (Iparallel + 2*G*Iperpendicular), using a G factor = 1. Data was relativized to baseline (Only FITC-Streptavidin) and centered to 0 (r/r0-1). Data was fitted, through non-linear regression, to binding models in R.

References:

[38]	W. Stöber, A. Fink, E. Bohn, “Controlled growth of monodisperse silica spheres in the micron size range” *Journal of Colloid and Interface Science* **1968**, *26*, 62–69.

[39]	A. Abdul-Gader, A. J. Miles, B. A. Wallace, “A reference dataset for the analyses of membrane protein secondary structures and transmembrane residues using circular dichroism spectroscopy” *Bioinformatics* **2011**, *27*, 1630–1636.

# Supplementary figures and table

**Fig S1.** Complete CD spectra of different length alanine-rich peptides, [1], [2], [3], in solution (10 mM phosphate buffer pH 7) at increasing temperatures.

**Fig S2.** CD spectra of K^7^ peptide [3] in solution at different pH values.

**Fig S3.** SEM analysis of SNPs. Micrographs of core (A) and amino-functionalized (B) SNPs, with the corresponding particle size distributions (C, D) extracted from SEM analysis. The core SNPs exhibit an average apparent diameter of 54 ± 5 nm (n = 250). As samples were sputter-coated with a ~3 nm gold layer, the measured diameter includes contributions from the coating on both sides of the particles; after correction (~6 nm total), the actual particle diameter is 48 ± 5 nm, indicating a narrow size distribution. No significant change in particle size or morphology is observed after APTES functionalization. Scale bars represent 50 nm.

**Fig S4.** Immobilization efficiency of the K^7^ peptide [3] on SNPs at different pH values.

**Fig S5.** CD spectra of K^7^ peptide [3] in solution (normal) and mixed with 10 mg/mL SNPs (dashed) at different temperatures.

**Fig S6.** CD spectra (at 2 °C) of K^7^ peptide [3] immobilized on SNPs at different Ph values (left) and temperature (right). Once immobilized, the peptide-SNP particles were resuspended in phosphate buffer pH 7, then the CD was measured.

**Fig S7.** MALDI-TOF spectrum of K^7^ [3], non-immobilized peptide left (supernatant) after SiNP immobilization.

**Fig S8.** (A) Additional dialdehydes used to immobilize the K^7^ peptide [3] onto SNPs. (B) CD spectra of immobilized peptides at 2 °C (left) or melting curves following ellipticity at 222 nm (right).

**Fig S9.** CD spectra of *K^7^* peptide [3] and its variants, *K^3^-A-K^3^* [4], *K^2^-A^3^-K^2^* [5] & K-A-K-A-KA-K [6], in solution at different temperatures and at pH 7 (A) and pH 10.8 (B).

**Fig S10.** Ellipticity at 222 nm of K^7^ [3] and K^7^ variants, [4], [5], [6], as function of temperature.

**Fig S11.** Crystal structure of streptavidin bound to SPB-Tag2. Residues engineered and changed for lysine residues are highlighted in dark orange (in SBP-5K variant, [7], all are changed by Lys, whereas in SBP-3K variant, [8], only the extreme and middle positions are changed by Lys. In SBP-CTRL, [9], only the middle position is changed by Lys, so its immobilization in SNPs is possible).

**Fig S12.** CD spectra of engineered SBP peptide [7] vs control [9]. (A) In solution at different pHs and temperatures and (B) immobilized on SNPs.

**Fig S13.** Fluorescence anisotropy experiments assessing the binding of streptavidin to the engineered SBP-5K peptide [7] immobilized on SNPs at 25 °C (A), and 70 °C (B). The control version, SBP-CTRL [9] is also plotted for comparison.

**Fig S14.** K^2^ peptide, [1] (9-mer), MALDI-TOF spectra. Obtained m/z: 1047.35, Calculated: 1047.2.

**Fig S15.** K^5^ peptide, [2] (16-mer), MALDI-TOF spectra.Obtained m/z: 1715.9, Calculated: 1716.

**Fig S16.** Analysis of K^7^ peptide, [3] (27-mer). (A) MALDI-TOF spectra. Obtained m/z: 2783.67, Calculated: 2783.25. (B) UV (280 nm) traces of size exclusion chromatography (SEC). Retention time: 18.160 min, Calculated purity: 97.85%.

**Fig S17.** Analysis of K^3^-A-K^3^ peptide [4]. (A) MALDI-TOF spectra. Obtained m/z: 2726.49, Calculated: 2726.16. (B) UV (280 nm) traces of SEC. Retention time: 18.163 min, Calculated purity: 98.98%.

**Fig S18.** Analysis of K^2^-A^3^-K^2^ peptide [5]. (A) MALDI-TOF spectra. Obtained m/z: 2611.87, Calculated: 2611.97. (B) UV (280 nm) traces of SEC. Retention time: 19.553 min, Calculated purity: 100%.

**Fig S19.** Analysis of K-A-K-A-K-A-K peptide [6]. (A) MALDI-TOF spectra. Obtained m/z: 2611.73, Calculated: 2611.97. (B) UV (280 nm) traces of SEC. Retention time: 18.722 min, Calculated purity: 93.79%.

**Fig S20.** Analysis of SBP-5K peptide [7]. (A) MALDI-TOF spectra. Obtained m/z: 2958.99, Calculated: 2957.52. (B) UV (214 nm) traces of SEC. Retention time: 17.812 min, Calculated purity: 96.40%.

**Fig S21.** Analysis of SBP-3K peptide [8]. (A) MALDI-TOF spectra. Obtained m/z: 2828.61, Calculated: 2828.2. (B) UV (214 nm) traces of SEC. Retention time: 17.973 min, Calculated purity: 99.05%.

**Fig S22.** Analysis of SBP-CTRL peptide [9]. (A) MALDI-TOF spectra. Obtained m/z: 2759.18, Calculated: 2759.10. (B) UV (214 nm) traces of SEC. Retention time: 18.106 min, Calculated purity: 94.67%.

**Table S1.** Proportion of alpha-helix based on CD spectra processing with SELCON3 algorithm. Based on mean residue ellipticity (RSE) on the 195-260 nm range using SMP_AU-PCDDB.
